# Impact of Daily and Seasonal Variation on the Phytochemical Profile of *Larrea cuneifolia* in Northwestern Argentina

**DOI:** 10.3390/plants14213332

**Published:** 2025-10-31

**Authors:** María Celeste Barrera, Mariana Daniela Rosa, Iris Catiana Zampini, María Inés Isla

**Affiliations:** 1 Instituto de Bioprospección y Fisiología Vegetal (INBIOFIV-CONICET-UNT), San Martín 1545, San Miguel de Tucumán T4000CBG, Argentina; mariacelestebarrera92@gmail.com (M.C.B.); zampini@csnat.unt.edu.ar (I.C.Z.); 2 Facultad de Ciencias Naturales, Instituto Miguel Lillo, Miguel Lillo 205, San Miguel de Tucumán T4000CBG, Argentina

**Keywords:** *Larrea cuneifolia*, seasonal and daily variation, total phenolic compounds, total flavonoids, nordihydroguaiaretic acid, antioxidant capacity

## Abstract

*Larrea cuneifolia* Cav. (common name: jarilla macho) is an endemic Argentinian medicinal shrub that has traditionally been used by the Diaguita-Calchaquí communities in the Monte Desert region in northwestern Argentina. The aim of the present study was to analyze the phytochemical profile and biological activity of the aerial parts of jarilla collected in different places throughout the year, in different seasons and times of day, to determine the optimal harvesting conditions for promoting its medicinal use. The aerial parts were collected three times a day over the course of four seasons in eight *L*. *cuneifolia* populations. The total phenolic compounds (TPCs), total flavonoid (TF) content, total lignans (TL), sugars (S) and soluble protein (SP) content were quantified by using spectrophotometric methods and HPLC-DAD. Antioxidant activity was determined by using ABTS scavenging. Significant seasonal, diurnal and spatial variations in the accumulation of TPC (52.61 to 113.52 mg GAE/g), TF (3.71 to 17.92 mg QE/g), TL (283 to 582 μg NDHGAE/g); S (5.73 to 15.17 mg GE/g) and SP (36.75 to 103.10 mg BSAE/g) in aerial parts of *L. cuneifolia* were revealed. The highest concentrations of TPC and TF were recorded in spring mornings. Maximum accumulation of nordihydroguaiaretic acid (291.8 ± 2.8 μg NDHGAE/mg dry weight) and other lignans were also observed in spring. Heat map analyses pinpoint Ampimpa (Site 1) as a site for jarilla sustainable harvesting, balancing high metabolite content with population abundance, especially in spring, when the highest antioxidant activity (SC_50_ = 1.560 ± 0.021 μg GAE/mL) coincides with increased phenol levels. These studies highlight the importance of integrating ecological and phytochemical data to define harvesting strategies; collecting during spring mornings optimizes the yield of bioactive compounds, simultaneously minimizing ecological pressure. This study demonstrates how seasonal bioprospecting can inform pharmacological research and local development while safeguarding the endemic plant population.

## 1. Introduction

**Figure 1:**
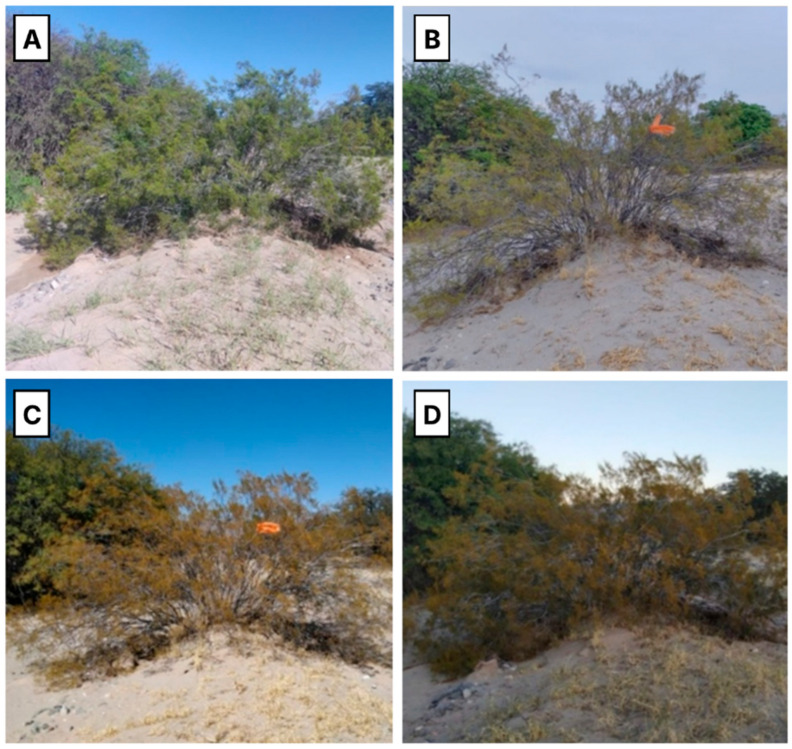
Photograph of *Larrea cuneifolia* in Monte region from Argentina. (**A**) summer (**B**) spring (**C**) winter (**D**) autumn.

A semiarid ecosystem, with relatively low humidity, temperature fluctuations and intense solar radiation [1], predominates in the eco-regions “Monte de Sierras y Bolsones” (between 1800 and 2700 masl) in north-western Argentina [1]. These regions, well-known for their great ecological, social, and cultural importance, are home to medicinal plant species that have been used since ancient times. Such is the case of *Larrea cuneifolia* Cav, a native shrub popularly known as jarilla, jarilla norte-sur or jarilla macho, which belongs to the family Zygophyllaceae (Figure 1) [2].

Due to limited access to urban areas and health centers, as well as the high cost of medicines, the indigenous community of the Calchaquí Valleys keeps a long-standing traditional practice of using *L. cuneifolia* as a remedy, especially when dealing with respiratory diseases, as well as musculoskeletal and skin disorders [1,3]. *L. cuneifolia* is recognized for its anti-inflammatory, antirheumatic, hypotensive, rubefacient, diaphoretic, febrifuge, oxytocic, emmenagogue, odontalgic and antitussive properties, as well as its antifungal properties [1,2,4,5]. In this sense, several studies have documented its anmicrobial activity against multi-resistant Gram-positive and Gram-negative bacteria [6], antifungal activity against phytopathogenic filamentous fungi and yeasts [7,8,9,10], larvicidal activity [11], antioxidant, antiinflammatory [3,12,13,14] and antitumoral [15] properties. Phytochemical studies revealed that this species accumulates bioactive polyphenol compounds [3,6,7,8,15]. It is important to note that, in addition to the traditional use that local communities make of *L. divaricata*, it is exploited directly from the environment by wild harvesting for the development of cosmetic commercial products for dandruff and hair loss [16,17]. These extractive practices can have negative effects on population abundance, so it is necessary to consider designing conservation methods aimed at maintaining the population abundance of the species. Furthermore, *L. cuneifolia* is on the red list of endemic plants in Argentina’s Monte Desert due to the intensive extraction from its natural habitat.

Biosynthesis and accumulation of metabolites in plants are regulated by multifactorial interactions between genetic and external environmental factors, i.e., temperature, light, humidity and precipitation, as well as circadian rhythms [18,19,20,21,22,23,24]. Control of primary and secondary metabolism ensures that phytochemicals are in tune with the demands of the environment and the available resources. Consequently, phytochemical composition varies throughout the day and year (seasonal variation) [18,19,20,21,22].

At present, both an increase in temperature and a progressive rainfall decrease have been observed in areas such as the Monte Desert, a fact which is starting to have a negative impact on the habitat of *L. cuneifolia* [23]. As metabolite production depends on environmental factors, these variations could also influence the phytochemical profile of *L. cuneifolia* and consequently affect its therapeutic value. A previous report described the distribution and population abundance of *L. cuneifolia* in the Calchaquies Valleys [1]. To promote sustainable harvesting of this species in this region, it is essential to conduct studies to identify not only the population but also the most appropriate season and time of day. It is the aim of the present study to determine the optimal conditions for harvesting this species for medicinal use.

## 2. Results and Discussion

**Figure 2:**
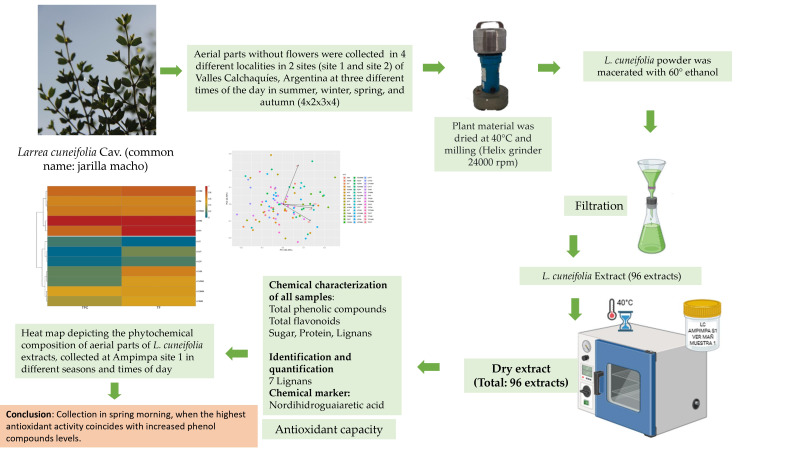
Flowchart of procedure to obtain and characterize *L. cuneifolia* extracts.

The Valley Calchaquies shrub lands are arid regions with daily and seasonal temperature variations and with seasonal rainfalls not exceeding 50 mm per year, generally concentrated in spring The aerial parts of *L. cuneifolia* were collected at three different times of the day in summer, winter, spring, and autumn (Figure 1). Then, they were dried and milled and 96 ethanolic extracts from the aerial parts were prepared. Both the yield of ethanol-soluble principles and the content of primary and specialized metabolites in *L. cuneifolia,* as well as the antioxidant activity, were determined (Figure 2).

### 2.1. Phytochemical Characterization

The yield of the soluble principles ranged from 0.1837 to 0.3974 g DW (dry weight of soluble principle)/g of dry plant material while the TPC content ranged from 52.61 to 113.52 mg GAE/g of dry plant material. TF content varied from 3.71 to 17.92 mg QE/g dry plant material depending on the time of day, season, and collection site. Variation was also observed in the content of primary metabolites, including reducing sugars (5.73 to 15.17 mg GE/g dry plant material) and soluble proteins (36.75 to 103.10 mg BSAE/g dry plant material). Seasonal and daily fluctuations of the metabolite content of *L. cuneifolia* collected in different sites are shown in Supplementary Material Tables S1–S5.

To integrate all data, i.e., location and collection site, time of year and the most appropriate time for collection, a heat map was drawn. The rows depict different locations and collection sites, seasons and times of day for harvesting and the columns depict the chemical composition in each one. The results were conveyed by using a color scale, where red indicates an increase in phytochemical concentration and blue indicates a decrease.

Cluster analyses based on Euclidean distance facilitated grouping of extracts according to their phytochemical composition, revealing distinct patterns within the clusters. Figure S1, shows two primary clusters. Cluster 1 includes extracts with high concentrations of TPC, TF, reducing sugars and soluble proteins (warm colors) and corresponds mostly to *L. cuneifolia* samples from Fuerte Quemado and Los Poleos, obtained in autumn and winter in the morning and the afternoon. Cluster 2 comprises samples with low and medium concentrations of TPC, TF, reducing sugars, and soluble proteins (cool colors). This group mainly comprises samples from Tío Punco and Ampimpa, obtained in summer and spring at midday.

The heatmap clearly illustrates variation in the phytochemical composition of *L. cuneifolia* extracts.

**Figure 3:**
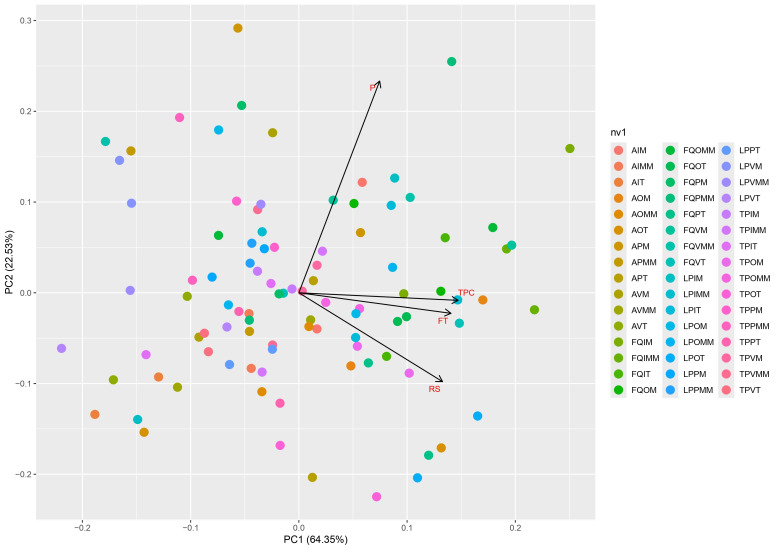
Principal Component Analysis (PCAs) of the phytochemical composition of aerial parts of *L. cuneifolia* collected at Ampimpa site 1 y2, Tío Punco site 1 y 2, Fuerte Quemado site 1 y 2, and Los Poleos site 1 y 2. A1, Ampimpa site 1; A2, Ampimpa site 2; TP1, Tío Punco site 1; TP2, Tío Punco site 2; FQ1, Fuerte Quemado site 1; FQ2, Fuerte Quemado site 2; LP1, Los Poleos site 1; LPS2, Los Poleos site 2; I, winter; O, autumn; P, spring; V, summer; M, morning; MM, midday; T, afternoon; TPC, total phenolic compounds; FT, total flavonoids; RS, reducing sugars; P, soluble proteins.

A principal component analysis (PCA) was performed because it provides valuable insights into the impact of individual variables on overall variability, which a heatmap alone cannot offer (Figure 3).

This analysis shows that two components explain a total cumulative variance of 86.88%. The first component (PC1) explains over 64% of the cumulative variance and is positively correlated with TPC and TF while the second component (PC2) explains 22.53% of the overall variance and is influenced by proteins and reducing sugar content. Points closest to TPC and TF correspond to FQ and LP sites with higher concentrations of TPC and TF (green and light blue points). TPC and TF content are the variables most strongly correlated with PC1, indicating that they play a key role in explaining overall variability among the samples. This suggests that extracts with higher concentrations of phenolic compounds and flavonoids will cluster on the positive side of PC1.

**Figure 4:**
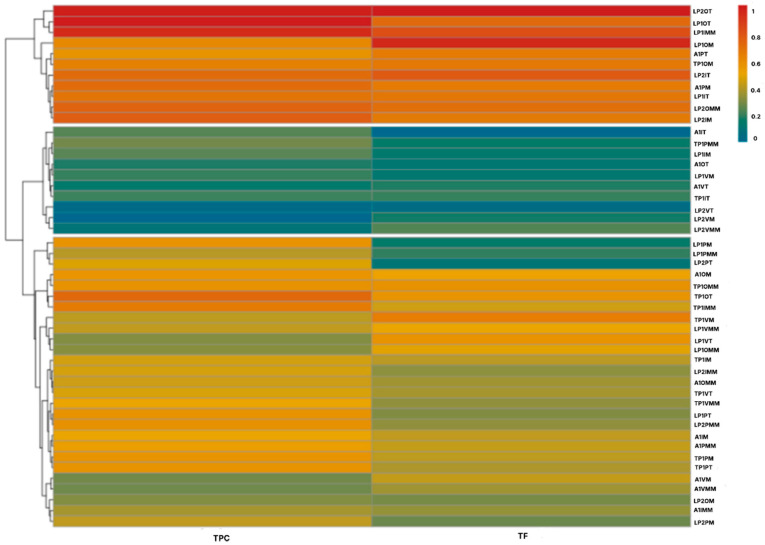
Heatmap depicting the phytochemical composition of aerial parts of *L. cuneifolia,* collected in A1, Ampimpa site 1; TP1, Tío Punco site 1; LP1, Los Poleos site 1; LPS2, Los Poleos site 2; I, winter; O, autumn; P, spring; V, summer; M, morning; MM, midday; T, afternoon; TPC, total phenolic compounds; TF, total flavonoids.

Although the plant material collected at Fuerte Quemado and Los Poleos showed the highest concentration of phytochemicals, previous studies have shown that these sites have a very low population abundance [1] making sustainable collection of plant material from these sites unviable. Conversely, a high biodiversity index value was found for *L. cuneifolia* at Ampimpa Site 2 [1], but this area is very likely to be affected by urbanistic development in the near future. Consequently, a heat map was made (Figure 4) that excluded these sites considering only samples from Los Poleos Sites 1 and 2, due to their high phytochemical content; the same criterion was used with Tío Punco Site 1 and Ampimpa Site 1, due to their higher population abundance [1].

Thus, three clusters were observed: Cluster 1 presents the warmest tones (red orange) and depicts samples with the highest TPC and TF concentrations collected at Los Poleos site 1 and 2 in autumn and winter, and at Ampimpa site 1 in spring. Cluster 2 corresponds to the coolest colors (green blue) and depicts the samples with the lowest concentration of TPC and FT, collected at the same spot in winter. Cluster 3 corresponds to *L. cuneifolia* extracts with average TPC and TF values of samples collected at Ampimpa site 1 during winter, autumn, and summer.

Based on these results, we conclude that Ampimpa Site 1 could be selected for sustainable harvesting, given the abundance of its population and the levels of TPC and TF present in plant tissue throughout the year, particularly in spring (Cluster 1).

**Figure 5:**
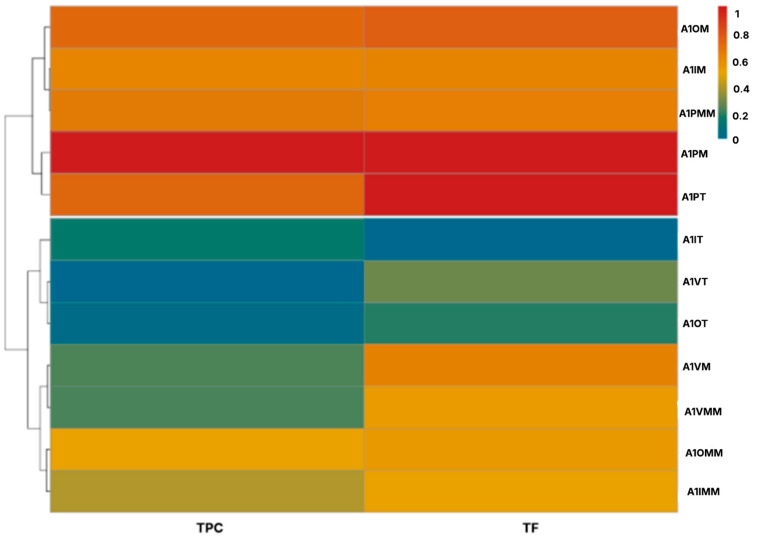
Heat map depicting the phytochemical composition of aerial parts of *L. cuneifolia* extracts, collected at Ampimpa site 1 in different seasons and times of day. A1, Ampimpa site 1; I, winter; O, autumn; P, spring; V, summer; M, morning; MM, midday; T, afternoon; TPC, total phenolic compounds; TF, total flavonoids.

To confirm this, a new heat map was drawn, this time considering only Ampimpa site 1 in all four seasons and at different times of day. Figure 5 shows this new heat map, which reveals two clusters. Cluster 1 includes samples with high phytochemical content from the morning, midday, and afternoon collections in spring, autumn, and winter, while Cluster 2 consists of extracts with medium or low phytochemical contents from the remaining seasons and periods. The analysis of the results in the present work indicates spring is the most appropriate season for collecting plant material, regardless of the time of day. Furthermore, the concentration of specialized metabolites observed in the morning has intermediate values in autumn and winter.

The variation in accumulation levels of phytochemicals in the plants sampled at different times of day and year can directly be affected by changes in light quality, temperature, and water availability both throughout the day and year [18,19,20]. Following are two examples of how light quality is closely related to those levels. The blue light and other shorter wavelengths are strongly affected by Rayleigh scattering; thus, a lower abundance of these wavelengths in winter as well as at dawn and dusk leads to an increase in the relative amount of the longer, red wavelengths. A second instance is *L. cuneifolia* paraheliotropism; linked to solar movements, it orients its leaves reversibly, thus allowing for greater use of solar energy in the early hours of the day [24,25]. In the Monte Desert in the Calchaquí Valleys, winter and autumn are dry seasons, rainfall < 50 mm, with shorter days and, consequently, fewer hours of sunlight [26]. Accordingly, our results show a decrease in the content of reducing sugars in winter and autumn evenings. This decrease in the levels of precursor molecules of specialized metabolites probably explains, in part, the observed decrease in TPC and TF contents observed for the said seasons and times (Tables S2–S4). Conversely, *Larrea divaricata,* another *Larrea* species that grows in Patagonia in the South of Argentina, shows a higher production of TPC and TF in autumn and a lower one in winter and spring [27]. The authors attribute this increase to mechanisms of resistance to water stress.

As previous studies have reported that this species primarily accumulates bioactive lignans [3,6,7,8,15], these compounds were quantified by using spectrophotometric methods, and HPLC-DAD on the extracts obtained from samples collected at Ampimpa S1. This was performed during the spring three times a day, and during the autumn and winter, in the morning, to determine the most suitable season to obtain lignan-rich extracts.

Total lignan quantification indicates that levels remain at around 497–582 µg NDHGAE/g dry weight of extract, except in *L. cuneifolia* samples collected in autumn and winter afternoons, when concentrations decrease by around 47% and 25%, respectively (Table 1). The expression of genes involved in the lignans biosynthesis pathway as well as in other phenolic compounds, is frequently altered by abiotic stress, light type and photoperiod regimes [18,19,20,28]. At the same time, lignans participate in the synthesis of other chemical components, resulting in a gradual decrease in their content in two seasons (autumn and winter) [29]. The lignans can contribute to the plant’s defense by being incorporated into cell walls, making them slightly more resistant to degradation, and reinforcing the plant’s structural barriers against pathogens and herbivores.

**Figure 6:**
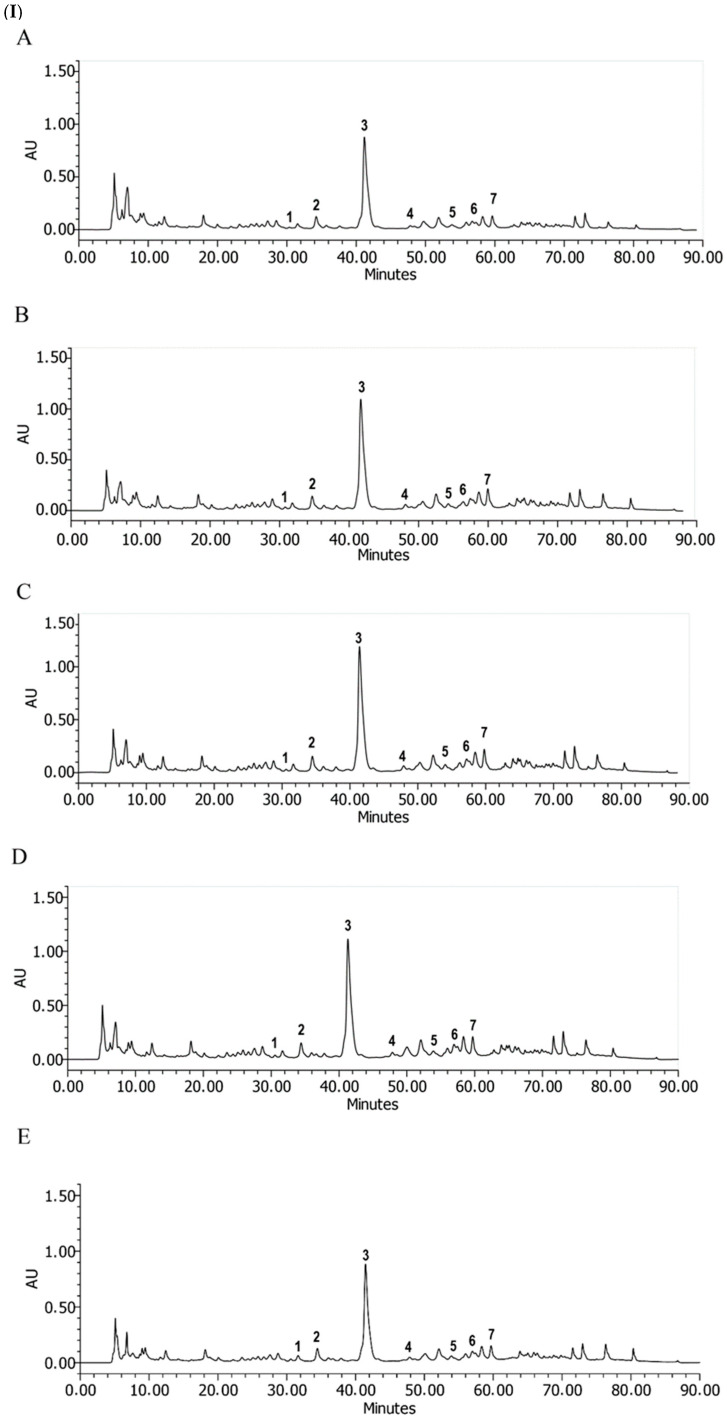

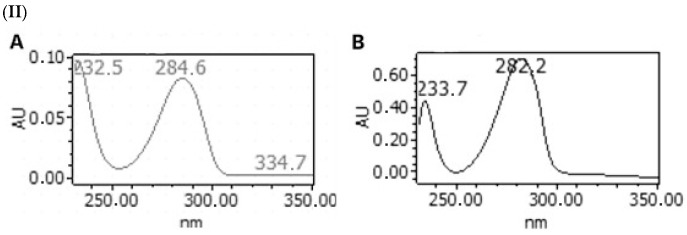
(**I**) HPLC profile of *L. cuneifolia* extracts, collected at Ampimpa S1 in (**A**) autumn/morning; (**B**) spring/morning; (**C**) spring/midday; (**D**) spring/afternoon; (**E**) winter/morning. **Peak 1:** 3,3′,4′-trihydroxy-4 methoxy 7,7′-epoxylignan; **peak 2:** 3,4,3′,4′-tetrahydroxy-6,7′ cyclolignan; **peak 3:** Nordihydroguaiaretic acid; **peak 4:** 3,3′-dihydroxy-4-methoxy 7,7′- epoxylignan; **peak 5:** 4-[4-(4-hydroxy-phenyl)-2,3 dimethyl-butyl]-benzene-1,2-diol; **peak 6:** Methyl nordihydroguaiaretic acid; **peak 7:** Methyl nordihydroguaiaretic acid isomer. (**II**) UV absorption spectra (196–375 nm) of peak 2 (**A**) and peak 3 (**B**).

HPLC-DAD profiles (Figure 6, Table 2) revealed the presence of seven lignans, including NDHGA, epoxylignans, and cyclolignans. NDHGA was more abundant in spring regardless of the time of day (254–292 μg/mg DW). However, a maximum accumulation of other lignans, such as 3,4,3′,4′-tetrahydroxy-6,7′ cyclolignan and 4-[4-(4-hydroxyphenyl)-2,3 dimethyl butyl [benzene-1,2-diol] was also observed in the morning. Based on these results, the most suitable conditions for a sustainable harvest of *L. cuneifolia* appear to be in spring, preferably in the morning.

The presence of high levels of NDGA and its derivatives confirms the medicinal importance of this species, as it exhibits chemopreventive and antimicrobial activities, effects on fertility and reproduction, as well as hypoglycemic activity, larvicidal activity and activity against *Botrytis cinerea* [12,30,31,32,33,34,35,36]. Antifungal and antioxidant activity has been previously described for ethanolic extracts of *L. cuneifolia* [7,12,13,14].

### 2.2. Biological Activity

The antioxidant activity of ethanolic extracts of *L. cuneifolia* from the Ampimpa site 1 location was found to have scavenging concentration values of 50% ABTS (SC_50_ values) ranging from 1.56 to 2.32 µg GAE/mL in samples collected during summer months and spring morning. In contrast, during winter and autumn, the SC_50_ values ranged from 3.07 to 4.9 µg GAE/mL (Table 3). The higher antioxidant capacity was recorded in spring (morning and midday). These results coincide with a higher NDHGA content and surpass the values reported by Moreno Et Al. (2018) [7].

## 3. Materials and Methods

### 3.1. Chemicals, Reagents and Materials

Ethanol was purchased from Cicarelli (Santa Fé, Argentina). Folin–Ciocalteau reagent, AlCl_3_, gallic acid, quercetin, 2,2′-Azino-bis (3-ethylbenzothiazoline-6-sulfonic acid) diammonium salt (ABTS), glucose, bovine serum albumin and HPLC grade solvent and standards were obtained from Sigma Aldrich, St. Louis, MO, USA.

### 3.2. Plant Material

**Figure 7:**
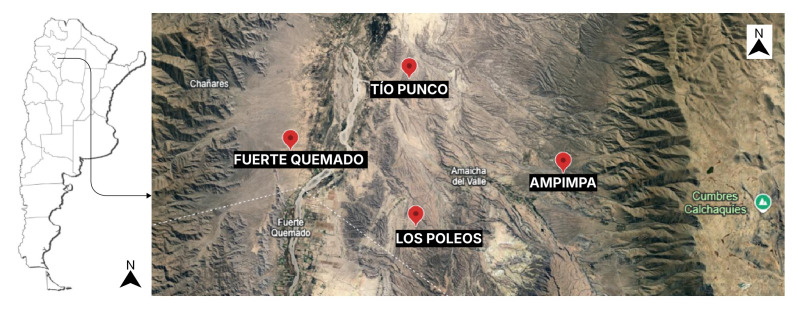
Locations of *L. cuneifolia* harvesting in Valles Calchaquies, Tucumán, Argentina.

Aerial parts of *L. cuneifolia* (Figure 1) were collected in the summer, autumn, winter and spring of 2022 and 2023, in the morning (8 a.m.), midday (1 p.m.) and afternoon (6 p.m.) at different locations: Ampimpa Site 1 (26°35′33″ S 65°55′00″ W; 1960 m a.s.l), Ampimpa Site 2 (26°35′23″ S 65°53′14″ W; 2109 m a.s.l), Tío Punco Site 1 (26°31′58″ S 65°57′53″ W; 1823 m a.s.l), Tío Punco Site 2 (26°31′34″ S 65°57′41″ W; 1816 m a.s.l), Fuerte Quemado Site 1 (26°34′31″ S 66°03′00″ W; 1855 m a.s.l), Fuerte Quemado Sitio2 (26°34′46″ S 66°03′27″ W; 1866 m a.s.l), Los Poleos Sitios1 (26°37′33″ S 65°57′33″ W; 1954 m a.s.l) and Los Poleos Sitio 2 (26°37′52″ S 65°57′52″ W; 1949 m a.s.l), Tucumán, Argentina (Figure 7). A field identification was performed by the authors using fresh material and native flora guide [37]. The determinations were subsequently confirmed by experts from the Phanerogamic Herbarium of the Miguel Lillo Foundation (LIL), San Miguel de Tucumán, Argentina [1].

Five plots were defined at each site, and three mid-canopy samples, which were subsequently pooled for processing, were taken from individuals of similar size in each plot. Collecting was performed in summer, autumn, winter and spring three times a day (morning, midday and afternoon). This resulted in three samples from each season on each site (3 times during the day × 4 seasons × 8 sites). The samples were 96 altogether.

The samples were dried until constant weight was reached in a forced air oven (Dhacel, FD101) at 40 °C, after which they were ground in a helical mill (Numak, F 100 Power ½ hp-0.75 KW, Brusque, Brazil). The powder was vacuum-sealed and stored at room temperature to protect the plant material from moisture, as well as physical and microbial contamination. Control specimens were deposited in the herbarium of the Miguel Lillo Foundation, Tucumán, Argentina.

### 3.3. Extract Preparation

Ethanolic extracts were prepared by using 1 g of plant material (96 samples) in 20 mL of 60° ethanol. The mixture was then macerated in an ultrasonic bath (Ultrasonic Washer Arcano Model PS-10A, Shandong Arcano Ultrasonic Technology Co., Ltd., Jinan, China) for 30 min at 40 °C. The extracts were then vacuum-filtered and dried in a vacuum oven (Arcano DF2-6020, Cientifica Dominguez, San Miguel de Tucumán, Argentina), at 40 °C until they reached a constant weight. The dried extracts were stored at −20 °C until use (g DW or g dry weight of extract). All extracts were obtained in triplicate.

### 3.4. Phytochemical Analysis

#### 3.4.1. Quantification of Total Phenolic Compounds

The total phenolic compound (TPC) content of each sample (96 extracts) was determined by using the Folin–Ciocalteau reagent [38]. Samples (1 mL of different dilutions of each extract) were oxidized with 0.1 mL of Folin–Ciocalteau reagent (Sigma-Aldrich), and then the reaction was neutralized with 0.4 mL of 15.9% sodium carbonate and kept at room temperature for 20 min. Measurements were performed by using a UV-visible spectrophotometer (Jasco V-630 Thermo Fisher Scientific, Tokyo, Japan) at 765 nm. Gallic acid was used as the reference compound.

Measurements were performed in triplicate on all extracts, and the results were expressed as milligrams of gallic acid equivalent (mg GAE) per gram of dry plant material (mg GAE/g PM) (Sigma Aldrich, St. Louis, MO, USA). The chemical stability of the plant material was assessed throughout a three-year period.

#### 3.4.2. Quantification of Total Flavonoids

The total flavonoid (TF) content of each extract (96 extracts) was determined according to the AlCl_3_ method [39]. Samples (1 mL of different dilutions of each extract) were added with 1.5 mL of ethanol 96° and 0.05 mL of AlCl_3_ 5%. The reaction was kept at room temperature for 30 min. Measurements were performed using a UV-visible spectrophotometer (Jasco V-630 Thermo Fisher Scientific, Tokyo, Japan) at 425 nm. Quercetin was used as the reference compound (Sigma Aldrich, St. Louis, MO, USA). Measurements were performed in triplicate on all extracts, and the results were expressed as milligrams of quercetin equivalent (mg QE) per gram of dry plant material (mg QE/g PM). The chemical stability of the plant material was assessed throughout a three-year period.

#### 3.4.3. Quantification of Total Lignans

To determine total lignan (TL) content, appropriate dilutions of 2 mL of each *L. cuneifolia* extract were performed, and their absorbance was read at 282 nm by using a UV-visible spectrophotometer (Jasco V-630 Thermo Fisher Scientific, Tokyo, Japan). A calibration curve of nordihydroguaiaretic acid (NDGA) (Sigma Aldrich, St. Louis, MO, USA) was drawn. The results were expressed as milligrams of nordihydroguaiaretic acid equivalents per gram of dry plant material (µg NDGAE/g PM). The chemical stability of the plant material was assessed throughout a three-year period.

### 3.5. Chromatographic Profile, HPLC-DAD

*L. cuneifolia* extracts were analyzed by means of high-performance liquid chromatography (HPLC), using a system including a Waters 1525 binary pump and a manual injection valve (Rheodyne Inc., Cotati, CA, USA) with a 20 μL loop. The separation was performed on a Waters C18, 5 μ, 4.6 × 250 mm column, kept at 25 °C. Analyses were performed by using a linear gradient composed of 1% formic acid in water (A) and acetonitrile (B) as follows: 30% to 40% B over 35 min, increasing to 45% B at 50 min, 70% B at 70 min, 70% to 100% B from 70 to 80 min, 100% to 30% B from 80 to 85 min, and keeping the 30% until the end at 95 min. The flow rate was 0.5 mL/min and the injected volume was 20 µL. Compounds were monitored at 280 nm. Compounds present in the extracts were identified by comparing retention times and UV-visible profiles with those of commercial reference compounds. Quantification of the different compounds was performed in NDGA equivalent to 280 nm. The chemical stability of the plant material was assessed throughout a three-year period.

### 3.6. Macronutrient Quantification

#### 3.6.1. Quantification of Reducing Sugars

Reducing the sugar content of each extract (96 extracts) was quantified by using the Somogyi-Nelson method [40,41]. Aliquots of each sample in a final volume of 500 µL were added to 500 µL of Somogyi’s reagent (1 part of 10% CuSO_4_.5H_2_O plus 9 parts of Na+ and K+ tartrate solution). The mixture was incubated at 100 °C for 15 min. Then, 500 µL of Nelson’s reagent (a solution of ammonium molybdate and disodium arsenate in sulfuric acid) and 1000 µL of distilled water were added. The absorbance was measured in a UV-Visible spectrophotometer (Jasco V-630 Thermo Fisher Scientific, Tokyo, Japan) at 520 nm. The reference standard curve was prepared with different concentrations of 1 mM glucose. Measurements were performed in triplicate and expressed as milligram glucose equivalent (GE) per gram of dry plant material (mg GE/g PM). The chemical stability of the plant material was assessed throughout a three-year period.

#### 3.6.2. Quantification of Soluble Proteins

Proteins were quantified by using the Bradford method [42]. Appropriate volumes of each extract were diluted in distilled water up to 800 µL. Then, 200 µL of Bradford’s reagent was added and incubated for 10 min at room temperature. Measurements were performed with a UV-visible spectrophotometer (Jasco V-630 Thermo Fisher Scientific, Tokyo, Japan) at 595 nm. Bovine serum albumin (BSA) was used as the reference compound. Measurements were performed in triplicate on all extracts (96 extracts) and expressed as milligrams of BSA equivalent per gram of dry plant material (mg BSAE/g PM). The chemical stability of the plant material was assessed throughout a three-year period.

### 3.7. Determination of Antioxidant Activity

The antioxidant activity of the ethanolic extracts of *L. cuneifolia* was assessed by using the ABTS (2,2-azinobis-(3-ethylbenzothioazolin-6-sulfonic acid) scavenging method (Sigma Aldrich, St. Louis, MO, USA) according to Re et al. (1999) [43]. The ABTS^•+^ radical is generated by reacting 7 mM ABTS (2,2′-azinobis (3-ethylbenzothiazolinone-6-sulfonic acid) with 2.45 mM potassium persulfate and incubating at room temperature in the dark for 16 h. The ABTS^•+^ solution is diluted with 95% ethanol until an absorbance of 0.90 at 734 nm is reached.

For the reaction mixture, 100 μL of different dilutions of extracts were added to 200 μL of the working solution and mixed by shaking. The reaction was carried out at room temperature. Absorbance was recorded at 764 nm after 6 min (Thermo Scientific Multiskan GO, Vanta, Finland). Results are expressed as SC_50_ values (µg GAE/mL), defined as the concentration of phenolic compounds required to eliminate 50% of the ABTS free radicals. Quercetin was used as the reference compound.

### 3.8. Statistical Analysis

A one-way analysis of variance (ANOVA) was used to assess the significance of the influence of each independent variable separately (season and time of day), followed by Tukey’s post hoc test. All results were expressed as mean ± standard deviation. Differences between samples were considered statistically significant at *p* ≤ 0.05. Statistical analyses were performed by using InfoStat V1.1 [44]. A heatmap was drawn with Euclidean distance and Ward’s clustering algorithm on the standardized dataset, performed in R software 3.0.2 [45]. Principal component analysis (PCA) was also performed. The score plot was performed based on the first and second principal components (PCs).

## 4. Conclusions

Based on the results obtained, we can conclude that harvesting the aerial parts of *L. cuneifolia* in spring and especially during the early morning ensures the highest efficiency when extracting phytochemicals, particularly lignans, compounds recognized for their biological activity. Our results emphasize the importance of combining ecological and phytochemical data when devising sustainable harvesting strategies in fragile environments such as the Monte Desert in the Calchaquies Valleys.

## Figures and Tables

**Table 1:** Total lignan content in *L. cuneifolia* samples collected at Ampimpa site 1 at different times of the year (summer, autumn, winter and spring) and hours of the day (morning, midday and afternoon) by using a spectrophotometric method.

Total Lignans (µg NDGAE/g DW)				
	Summer	Autumn	Winter	Spring
Morning	518.5 ± 5.78 ^Ba^	496.60 ± 3.93 ^Ab^	511.24 ± 3.53 ^Bb^	517.84 ± 7.06 ^Ba^
Midday	558.7 ± 6.12 ^Cb^	533.24 ± 2.30 ^Bc^	519.73 ± 4.63 ^Ab^	564.36 ± 6.11 ^Cb^
Afternoon	517.42 ± 2.61 ^Ca^	282.59 ± 5.79 ^Aa^	384.89 ± 4.81 ^Ba^	581.84 ± 3.12 ^Db^

NDGAE, nordihydroguaiaretic acid equivalent; DW, dry weight of extract. Data were analyzed by one-way ANOVA for each factor (season and time of day) independently, followed by Tukey’s multiple comparison test (*p* ≤ 0.05). Different capital letters in the same line indicate significant differences between seasons, and lowercase letters indicate significant differences between times of day.

**Table 2:** Quantification of the different compounds identified in the extracts of *L. cuneifolia* from samples collected in autumn, spring and winter at different times of the day using HPLC-DAD.

Compounds			Autumn	Spring			Winter
			Morning	Morning	Midday	Afternoon	Morning
		RT. (Minutes)	A	B	C	D	E
			µg of Lignans in NDGA Equivalent/mg of Soluble Principle (Dry Weight of Extract)				
1	3,3′,4′-trihydroxy-4 methoxy 7,7′-epoxylignan	31.5	10.2 ± 4.7	10.1 ± 4.8	9.9 ± 1.6	11.8 ± 7.9	8.5 ± 5.5
2	3,4,3′,4′-tetrahydroxy-6,7′ cyclolignan	34.2	21.2 ± 3.6	73.0 ± 0.2	20.8 ± 0.0	24.6 ± 2.0	19.5 ± 5.2
3	Nordihydroguaiaretic acid	41.19	182.1 ± 2.9	273.4 ± 2.5	291.8 ± 2.8	253.6 ± 3.4	187.6 ± 3.1
4	3,3′-dihydroxy-4-methoxy 7,7′- epoxylignan	47.7	4.2 ± 0.3	8.6 ± 0.3	9.3 ± 2.5	8.8 ± 4.8	6.5 ± 0.5
5	4-[4-(4-hydroxy-phenyl)-2,3 dimethyl-butyl]-benzene-1,2-diol	53.7	10.1 ± 1.4	40.6 ± 4.2	14.6 ± 1.7	13.6 ± 1.3	8.3 ± 5.2
6	Methyl nordihydroguaiaretic acid	55.8	12.8 ± 3.6	14.8 ± 3.9	18.2 ± 5.1	17.5 ± 1.1	10.8 ± 0.5
7	Methyl nordihydroguaiaretic acid isomer	59.6	20.5 ± 0.5	28.4 ± 5.2	33.4 ± 1.3	30.9 ± 3.0	21.3 ± 3.7

RT, retention time; **A:** autumn/morning extract; **B:** spring/morning extract; **C:** spring/midday extract; **D:** spring/afternoon extract; **E:** winter/morning extract; NDGA, nordihydroguaiaretic acid.

**Table 3:** Antioxidant activity of extracts obtained from plant material from the Ampimpa Site, at different times of the year (summer, autumn, winter and spring) and hours of the day (morning, midday and afternoon).

SC_50_ (µg GAE/mL)				
	Summer	Autumn	Winter	Spring
Morning	2.323 ± 0.012 ^Ba^	3.838 ± 0.122 ^Db^	3.227 ± 0.011 ^Cb^	1.560 ± 0.021 ^Aa^
Midday	2.250 ± 0.009 ^Ba^	3.434 ± 0.009 ^Da^	3.071 ± 0.023 ^Ca^	1.647 ± 0.155 ^Aa^
Afternoon	2.299 ± 0.092 ^Aa^	4.977 ± 0.043 ^Cc^	3.644 ± 0.072 ^Bc^	2.116 ± 0.185 ^Ab^

GAE, gallic acid equivalent; Data were analyzed by one-way ANOVA for each factor (season and time of day) independently, followed by Tukey’s multiple comparison test (*p* ≤ 0.05). Different capital letters in the same line indicate significant differences between seasons, and lowercase letters indicate significant differences between times of day.

## Data Availability

The original contributions presented in this study are included in the article/Supplementary Material. Further inquiries can be directed to the corresponding authors.

## Supplementary Materials

The following supporting information can be downloaded at: https://www.mdpi.com/article/10.3390/plants14213332/s1, Table S1. Content of soluble principles in ethanol. Table S2. Phenolic compound content in *L. cuneifolia* samples collected in different seasons and at different times of the day. Table S3. Flavonoid content in *L. cuneifolia* samples collected in different seasons and at different times of the day. Table S4. Content of reducing sugars in samples of *L. cuneifolia* collected in different seasons and at different times of the day. Table S5. Protein content in *L. cuneifolia* samples collected in different seasons and at different times of the day. Figure S1. Heat map of the phytochemical composition of *L. cuneifolia* aerial parts collected at different sites. locations. seasons and times of day.

## References

[1] Barrera M.C., Kobak C.P., Uriburu F.M.C., Cuello A.S., Perea M.C., Torres S., Zampini C.I., Isla M.I., Rosa M.D. (2025). Patterns of diversity of medicinal shrubs in the Monte desert in the Calchaquí Valleys (Tucumán, Argentina). Lilloa.

[2] Ceballos S.J., Perea M.C. (2014). Plantas medicinales utilizadas por la comunidad indígena de Quilmes (Tucumán, Argentina). Bol. Latinoam. Caribe Plantas Med. Aromat..

[3] Carabajal M.P.A., Perea M.C., Isla M.I., Zampini I.C. (2020). The use of jarilla native plants in a Diaguita-Calchaquí indigenous community from northwestern Argentina: An ethnobotanical, phytochemical and biological approach. J. Ethnopharm..

[4] Barboza G., Cantero J., Nuñez C., Pacciaroni A., Ariza Espinar L. (2009). Medicinal plants: A general review and a phytochemical and ethnopharmacological screening of the native Argentine flora. Kurtziana.

[5] Ladio A.H., Gómez D.D. (2006). Plantas medicinales en comunidades rurales de Argentina: Usos, conocimiento y manejo. Rev. Argent. Cienc. Nat..

[6] Conta A., Simirgiotis M.J., Martínez Chamás J., Isla M.I., Zampini I.C. (2025). Extraction of Bioactive Compounds from *Larrea cuneifolia* Cav. Using Natural Deep Eutectic Solvents: A Contribution to the Plant Green Extract Validation of Its Pharmacological Potential. Plants.

[7] Moreno M.A., Córdoba S., Zampini I.C., Mercado M.I., Ponessa G., Sayago J.E., Pino Ramos L.L., Schmeda-Hirschmanne G., Isla M.I. (2018). Argentinean *Larrea* dry extracts with potential use in vaginal candidiasis. Nat. prod. commun..

[8] Moreno M.A., Zampini I.C., Isla M.I. (2020). Antifungal, anti-inflammatory and antioxidant activity of bi-herbal mixtures with medicinal plants from Argentinean highlands. J. Ethnopharmacol..

[9] Quiroga E.M., Sampietro A.R., Vattuone M.A. (2001). Screening antifungal activities of selected medicinal plants. J. Ethnopharmacol..

[10] Svetaz L., Zuljan F., Derita M., Petenatti E., Tamayo G., Cáceres A., Cechinel Filho V., Giménez A., Pinzón R., Zacchino S.A. (2010). Value of the ethnomedical information for the discovery of plants with antifungal properties. A survey among seven Latin American countries. J. Ethnopharmacol..

[11] Batallán G., Torre R., Flores F., Konigheim B., Ludueña-Almeida F., Tonn C., Contagiani M., Almirón W. (2013). Larvicidal activity of crude extracts from *Larrea cuneifolia* (Zygophyllaceae) and of its metabolite nordihydroguaiaretic acid against the vector *Culex quinquefasciatus* (Diptera: Culicidae). Rev. Soc. Bras. Med. Trop..

[12] Carabajal M.P.A., Isla M.I., Zampini I.C. (2017). Evaluation of antioxidant and antimutagenic activity of herbal teas from native plants used in traditional medicine in Argentina. S. Afr. J. Botany..

[13] Lorenzo M.E., Gómez P.E., Sabatino E., Segovia A.F., Figueroa L.C., Baroni M.V. (2020). Phenolic Profile and Antioxidant Activity of Ethanolic Extract of *Larrea cuneifolia* Cav. Leaves. Proceedings.

[14] Torres R., Urbina F., Morales C., Modak B., Delle Monache F. (2003). Antioxidant properties of lignans and ferulic acid from the resinous exudate of *Larrea nitida*. J. Chil. Chem. Soc..

[15] Carabajal M.P.A., Piloto-Ferrer J., Nicollela H.D., Squarisi I.S., Guissone A.P.P., Esperandim T.R., Crispim Tavares D., Isla M.I., Zampini I.C. (2021). Antigenotoxic, antiproliferative and antimetastatic properties of a combination of native medicinal plants from Argentina. J. Ethnopharm..

[16] Davicino R., Alonso R., Anesini C. (2010). Activity of a combination of decaffeinated coffee and *Larrea divaricata* Cav. aqueous extract on hair growth. Pat. Bulletin..

[17] Iannicelli J., Guariniello J., Pitta Alvarez S.I., Escandón A.S. (2018). Traditional uses, conservation estatus and biotechnological advances for a group of aromatic/medicinal native plants from America. Boletín Latinoam. Caribe Plantas Med. Aromáticas.

[18] Soni U., Brar S., Gauttam V.K. (2015). Effect of seasonal variation on secondary metabolites of medicinal plants. Int. J. Pharm. Sci. Res..

[19] Liebelt D.J., Jordan J.T., Doherty C.J. (2019). Only a matter of time: The impact of daily and seasonal rhythms on phytochemicals. Phytochem. Rev..

[20] Ramakrishna A., Ravishankar G.A. (2011). Influence of abiotic stress signals on secondary metabolites in plants. Plant Signal Behav..

[21] Thakur M., Bhattacharya S., Khosla P.K., Puri S. (2019). Improving production of plant secondary metabolites through biotic and abiotic elicitation. JARMAP.

[22] Ahmad I., Ahmad M.S.A., Ashraf M., Hussain M., Ashraf M.Y. (2011). Seasonal variation in some medicinal and biochemical ingredients in *Mentha longifolia* (L.) *Huds*. Pak J Bot..

[23] Souto C.P., Zalazar L.P., Tadey M., Premoli A.C. (2024). Modeling past, present and future: Species-specific responses to climate changes in three shrub congeners from South American drylands. J. Arid. Environ..

[24] Diaz S.M., Mangione A.M. (2015). Paraheliotropismo en hojas de Jarilla Norte-Sur (*Larrea cuneifolia* Cav.). Métodos Ecol. Sistemática..

[25] Medeiros J.S., Begaye A., Hanson D.T., Logan B., Pockman W.T. (2015). Photoprotective response to chilling differs among high and low latitude *Larrea divaricata* grown in a common garden *J*. Arid. Environ..

[26] https://www.meteoblue.com/es/tiempo/historyclimate/climatemodelled/amaicha-del-valle_argentina_3865969.

[27] Varela M.C., Arslan I., Reginato M.A., Cenzano A.M., Luna M.V. (2016). Phenolic compounds as indicators of drought resistance in shrubs from Patagonian shrublands (Argentina). Plant Physiol. Biochem..

[28] Singh R., Iqbal N., Umar S., Ahmad S. (2024). Lignan Enhancement: An Updated Review on the Significance of Lignan and Its Improved Production in Crop Plants. Phyton.

[29] Zitong Z., Manqun L., Binhong Z., Zhang F., Peijin N., Zhang Z., Sun D., Wang Z., Shi G., Ai J. (2025). Dynamic Changes in Lignan Content and Antioxidant Capacity During the Development of Three Cultivars of Schisandra chinensis Seeds. Horticulturae.

[30] Boiteux J., Espino M., Fernández M.D.L.Á., Pizzuolo P., Silva M.F. (2019). Control sustentable poscosecha: Extractos de *Larrea cuneifolia* mediados por nades frente a *Botrytis cinerea*. Rev. Fac. Cienc. Agrar. Univ. Nac. Cuyo..

[31] Craigo J., Callahan M., Huang R.C., DeLucia A.L. (2000). Inhibition of human papillomavirus type 16 gene expression by nordihydroguaiaretic acid plant lignan derivatives. Antivir. Res..

[32] Heller J.D., Kuo J., Wu T.C., Kast W.M., Huang R.C. (2001). Tetra O-methyl nordihydroguaiaretic acid induces G2 arrest in mammalian cells and exhibits tumoricidal activity in vivo. Cancer Res..

[33] Lambert J.D., Dorr R., Timmermann B.N. (2004). Nordihydroguaiaretic acid: A review of its numerous and varied biological activities. Pharm. Biol..

[34] Lambert J.D., Meyers R.O., Timmermann B.N., Dorr R.T. (2001). tetra-O-Methylnordihydroguaiaretic acid inhibits melanoma in vivo. Cancer Lett..

[35] Saleem M., Kim H.J., Ali M.S., Lee Y.S. (2005). An update on bioactive plant lignans. Nat. Prod. Rep..

[36] Scribner K., Gadbois T., Gowri M., Azhar S., Reaven G.M. (2000). Masoprocol decreases serum triglyceride concentrations in rats with fructose-induced hypertriglyceridemia. Metab. Clin. Exp..

[37] Carrizo J., Grau A. (2014). Plantas Silvestres de los Valles Calchaquíes.

[38] Singleton V.L., Orthofer R., Lamuela-Raventos R.M. (1999). Analysis of total phenols and other oxidation substrates and antioxidants by means of Folin Ciocalteu reagent. Meth. Enzymol..

[39] Woisky R.G., Salatino A. (1998). Analysis of propolis: Some parameters and procedures for chemical quality control. J. Apic. Res..

[40] Somogyi M. (1945). A new reagent for the determination of sugars. J. Biol. Chem..

[41] Nelson N. (1944). A photometric adaptation of the Somogyi method for the determination of glucose. J. Biol. Chem..

[42] Bradford M.M. (1976). A rapid and sensitive method for the quantitation of microgram quantities of protein utilizing the principle of protein-dye binding. Anal. Biochem..

[43] Re R., Pellegrini N., Proteggente A., Pannala A., Yang M., Rice-Evans C. (1999). Antioxidant activity applying an improved ABTS radical cation decolorization assay. Free Radic. Biol. Med..

[44] Di Rienzo J.A., Casanoves F., Balzarini M.G., Gonzalez L., Tablada M., Robledo C.W. (2012). InfoStat Versión 2015.

[45] RStudio Team (2020). RStudio: Integrated Development for R..

