# Supplementary material for: Impact of Daily and Seasonal Variation on the Phytochemical Profile of Larrea cuneifolia in Northwestern Argentina
Source: Plants (Basel). 2025 Oct 31;14(21):3332. doi: 10.3390/plants14213332 (PMC12608709; doi:10.3390/plants14213332)
Supplement: Supplementary file 1 [file plants-14-03332-s001.zip › plants-3908388-supplementary.pdf]

Supplementary material

Table S1. Content of soluble principles in ethanol.

**Soluble principle g SP/g PM**				
	Winter	autumn	Spring	Summer
**Ampimpa Site 1**				
Morning	0.306 ± 0.002 ^{Cb}	0.281 ± 0.002 ^{Bc}	0.305 ± 0.001 ^{Cab}	0.260 ± 0.002 ^{Aa}
Midday	0.286 ± 0.002 ^{Ba}	0.272 ± 0.001 ^{Ab}	0.303 ± 0.001 ^{Ca}	0.301 ± 0.002 ^{Cb}
Afternoon	0.283 ± 0.001 ^{Ba}	0.263 ± 0.001 ^{Aa}	0.309 ± 0.002 ^{Cb}	0.265 ± 0.003 ^{Aa}
**Ampimpa Site 2**				
Morning	0.332 ± 0.002 ^{Dc}	0.308 ± 0.002 ^{Bb}	0.322 ± 0.002 ^{Ca}	0.222 ± 0.001 ^{Aa}
Midday	0.311 ± 0.002 ^{Bb}	0.290 ± 0.002 ^{Aa}	0.319 ± 0.003 ^{Ca}	0.293 ± 0.002 ^{Ac}
Afternoon	0.302 ± 0.003 ^{Ba}	0.316 ± 0.003 ^{Bb}	0.356 ± 0.003 ^{Cb}	0.269 ± 0.012 ^{Ab}
**Tío Punco Site 1**				
Morning	0.298 ± 0.002 ^{BCa}	0.267 ± 0.002 ^{Aa}	0.299 ± 0.004 ^{Ca}	0.292 ± 0.002 ^{Ba}
Midday	0.326 ± 0.002 ^{Db}	0.274 ± 0.002 ^{Ab}	0.310 ± 0.002 ^{Cb}	0.292 ± 0.003 ^{Ba}
Afternoon	0.295 ± 0.002 ^{Aa}	0.315 ± 0.002 ^{Cc}	0.320 ± 0.003 ^{Cc}	0.300 ± 0.003 ^{Bb}
**Tío Punco Site 2**				
Morning	0.315 ± 0.002 ^{Cc}	0.305 ± 0.003 ^{Bb}	0.303 ± 0.002 ^{Ba}	0.272 ± 0.004 ^{Ab}
Midday	0.280 ± 0.004 ^{Ba}	0.293 ± 0.002 ^{Ca}	0.308 ± 0.002 ^{Da}	0.268 ± 0.003 ^{Aa}
Afternoon	0.297 ± 0.001 ^{Bb}	0.317 ± 0.003 ^{Cb}	0.326 ± 0.003 ^{Db}	0.268 ± 0.001 ^{Aa}
**Fuerte Quemado Site 1**				
Morning	0.367 ± 0.002 ^{Ba}	0.384 ± 0.002 ^{Cc}	0.367 ± 0.003 ^{Ba}	0.345 ± 0.002 ^{Ac}
Midday	0.393 ± 0.002 ^{Cb}	0.316 ± 0.002 ^{Ba}	0.397 ± 0.002 ^{Cc}	0.295 ± 0.003 ^{Aa}
Afternoon	0.364 ± 0.002 ^{Ca}	0.342 ± 0.002 ^{Bb}	0.386 ± 0.003 ^{Db}	0.314 ± 0.003 ^{Ab}
**Fuerte quemado Site 2**				
Morning	0.355 ± 0.002 ^{Da}	0.327 ± 0.003 ^{Bb}	0.347 ± 0.003 ^{Ca}	0.289 ± 0.004 ^{Aa}
Midday	0.372 ± 0.004 ^{Cb}	0.328 ± 0.003 ^{Bb}	0.374 ± 0.002 ^{Cb}	0.306 ± 0.003 ^{Ab}
Afternoon	0.352 ± 0.003 ^{Ca}	0.308 ± 0.003 ^{Aa}	0.380 ± 0.003 ^{Db}	0.323 ± 0.003 ^{Ac}
**Los Poleos Site 1**				
Morning	0.332 ± 0.002 ^{Ca}	0.372 ± 0.003 ^{Dc}	0.314 ± 0.003 ^{Ba}	0.228 ± 0.002 ^{Aa}
Midday	0.375 ± 0.002 ^{Dc}	0.289 ± 0.003 ^{Ba}	0.359 ± 0.003 ^{Cc}	0.234 ± 0.002 ^{Ab}
Afternoon	0.350 ± 0.004 ^{Db}	0.346 ± 0.002 ^{Cb}	0.323 ± 0.003 ^{Bb}	0.283 ± 0.003 ^{Ac}
**Los Poleos Site 2**				
Morning	0.359 ± 0.004 ^{Cc}	0.290 ± 0.003 ^{Ba}	0.371 ± 0.003 ^{Db}	0.183 ± 0.003 ^{Aa}
Midday	0.339 ± 0.004 ^{Bb}	0.326 ± 0.002 ^{Bb}	0.363 ± 0.004 ^{Ca}	0.210 ± 0.003 ^{Ab}
Afternoon	0.310 ± 0.003 ^{Ba}	0.351 ± 0.002 ^{Cc}	0.373 ± 0.002 ^{Db}	0.203 ± 0.003 ^{Ab}

µg SP/g PM: milligrams of soluble principles per gram of plant material. Data were analyzed by one-way ANOVA for each factor (season and time of day) independently, followed by Tukey's multiple comparison test ($p \leq 0.05$). Different capital letters in the same line indicate significant differences between seasons, and lowercase letters indicate significant differences between times of day.

Table S2. Phenolic compound content in *L. cuneifolia* samples collected in different seasons and at different times of the day.

Phenolic compound (mg GAE/g PM)				
	Winter	Autumn	Spring	Summer
**Ampimpa Site 1**				
Morning	79.59 ± 0.33 ^{Bc}	83.42 ± 0.33 ^{Cc}	93.61 ± 0.08 ^{Dc}	64.66 ± 0.50 ^{Ab}
Midday	70.82 ± 0.40 ^{Bb}	75.56 ± 0.42 ^{Cb}	81.23 ± 0.07 ^{Da}	64.50 ± 0.60 ^{Ab}
Afternoon	61.92 ± 0.51 ^{Ba}	57.64 ± 0.45 ^{Aa}	83.89 ± 0.60 ^{Cb}	56.41 ± 0.44 ^{Aa}
**Ampimpa Site 2**				
Morning	95.73 ± 0.27 ^{Cc}	99.45 ± 0.58 ^{Dc}	68.33 ± 0.26 ^{Bb}	60.66 ± 0.45 ^{Aa}
Midday	71.65 ± 0.57 ^{Bb}	80.25 ± 0.54 ^{Da}	58.98 ± 0.28 ^{Aa}	77.54 ± 0.60 ^{Cb}
Afternoon	64.15 ± 0.48 ^{Ba}	95.94 ± 0.24 ^{Db}	88.68 ± 0.28 ^{Cc}	61.24 ± 0.44 ^{Aa}
**Tío Punco Site 1**				
Morning	76.15 ± 0.23 ^{Bb}	88.56 ± 0.47 ^{Db}	83.66 ± 0.25 ^{Cb}	73.56 ± 0.22 ^{Aa}
Midday	89.79 ± 0.40 ^{Dc}	84.42 ± 0.14 ^{Ca}	64.56 ± 0.14 ^{Aa}	79.93 ± 0.66 ^{Bc}
Afternoon	60.00 ± 0.38 ^{Aa}	94.40 ± 0.70 ^{Dc}	83.82 ± 0.41 ^{Cb}	76.93 ± 0.84 ^{Bb}
**Tío Punco Site 2**				
Morning	81.90 ± 0.57 ^{Ac}	94.87 ± 0.16 ^{Cc}	83.32 ± 0.22 ^{Bc}	81.48 ± 0.68 ^{Ac}
Midday	66.27 ± 0.25 ^{Aa}	92.89 ± 0.23 ^{Db}	75.88 ± 0.08 ^{Ba}	78.10 ± 0.55 ^{Cb}
Afternoon	68.63 ± 0.20 ^{Bb}	76.15 ± 0.08 ^{Ca}	77.44 ± 0.19 ^{Db}	67.86 ± 0.13 ^{Aa}
**Fuerte Quemado Site 1**				
Morning	125.33 ± 0.44 ^{Dc}	115.58 ± 0.62 ^{Cc}	84.15 ± 0.27 ^{Aa}	85.45 ± 0.43 ^{Bb}
Midday	120.84 ± 0.10 ^{Db}	71.47 ± 0.50 ^{Ba}	87.73 ± 0.58 ^{Cb}	59.83 ± 0.47 ^{Aa}
Afternoon	112.04 ± 0.11 ^{Da}	105.72 ± 0.54 ^{Cb}	102.53 ± 0.32 ^{Bc}	90.82 ± 0.30 ^{Ac}
**Fuerte quemado Site 2**				
Morning	96.71 ± 0.24 ^{Bb}	101.86 ± 0.30 ^{Cb}	65.39 ± 0.56 ^{Aa}	104.85 ± 0.45 ^{Db}
Midday	109.85 ± 0.19 ^{Cc}	113.52 ± 0.10 ^{Dc}	106.64 ± 0.48 ^{Bb}	93.59 ± 0.64 ^{Aa}
Afternoon	93.27 ± 0.25 ^{Aa}	93.70 ± 0.27 A ^{Aa}	112.55 ± 0.47 ^{Cc}	106.72 ± 0.23 ^{Bc}
**Los Poleos Site 1**				
Morning	62.10 ± 0.30 ^{Ba}	86.61 ± 0.19 ^{Ba}	93.61 ± 0.08 ^{Ca}	72.91 ± 0.30 ^{Aa}
Midday	109.22 ± 0.33 ^{Cc}	67.40 ± 0.27 ^{Cc}	81.23 ± 0.07 ^{Bb}	84.17 ± 0.47 ^{Ac}
Afternoon	92.01 ± 0.47 ^{Cb}	112.55 ± 0.20 ^{Cb}	83.89 ± 0.60 ^{Bb}	84.18 ± 0.49 ^{Ab}
**Los Poleos Site 2**				
Morning	97.19 ± 0.40 ^{Db}	66.64 ± 0.20 ^{Ba}	73.74 ± 0.23 ^{Ca}	46.28 ± 0.14 ^{Aa}
Midday	76.40 ± 0.24 ^{Ba}	95.51 ± 0.56 ^{Db}	84.68 ± 0.30 ^{Cc}	52.61 ± 0.28 ^{Ac}
Afternoon	93.76 ± 0.09 ^{Cb}	111.70 ± 0.18 ^{Dc}	77.34 ± 0.34 ^{Bb}	47.84 ± 0.15 ^{Ab}

mg GAEg PM: milligrams of gallic acid equivalent per gram of plant material. Data were analyzed by one-way ANOVA for each factor (season and time of day) independently, followed by Tukey's multiple comparison test ($p \leq 0.05$). Different capital letters in the same line indicate significant differences between seasons, and lowercase letters indicate significant differences between times of day.

Table S3. Flavonoid content in *L. cuneifolia* samples collected in different seasons and at different times of the day.

Flavonoid (mg QE/g PM)				
	Winter	Autumn	Spring	Summer
**Ampimpa Site 1**				
Morning	7.90 ± 0.04 [Ac]	8.85 ± 0.02 [Bc]	10.36 ± 0.05 [Cb]	7.98 ± 0.05 [Ac]
Midday	7.14 ± 0.02 [Ab]	7.32 ± 0.04 [Bb]	8.02 ± 0.02 [Ca]	7.30 ± 0.07 [Bb]
Afternoon	3.71 ± 0.03 [Aa]	4.90 ± 0.03 [Ba]	10.36 ± 0.02 [Db]	5.50 ± 0.05 [Ca]
**Ampimpa Site 2**				
Morning	8.47 ± 0.06 [Bc]	10.43 ± 0.08 [Db]	5.02 ± 0.02 [Ab]	9.22 ± 0.03 [Cb]
Midday	6.63 ± 0.04 [Bb]	7.99 ± 0.03 [Ca]	3.93 ± 0.02 [Aa]	10.22 ± 0.04 [Dc]
Afternoon	4.45 ± 0.03 [Aa]	13.12 ± 0.11 [Dc]	9.07 ± 0.05 [Cc]	8.01 ± 0.02 [Ba]
**Tío Punco Site 1**				
Morning	7.82 ± 0.03 [Ab]	10.47 ± 0.07 [Cb]	7.89 ± 0.03 [Ac]	10.25 ± 0.04 [Bc]
Midday	8.20 ± 0.07 [Cc]	9.57 ± 0.05 [Da]	5.29 ± 0.04 [Aa]	7.03 ± 0.06 [Ba]
Afternoon	5.73 ± 0.05 [Aa]	9.41 ± 0.09 [Ca]	7.56 ± 0.03 [Bb]	7.44 ± 0.09 [Bb]
**Tío Punco Site 2**				
Morning	7.33 ± 0.05 [Ba]	11.76 ± 0.14 [Dc]	4.81 ± 0.06 [Aa]	9.38 ± 0.03 [Cc]
Midday	8.42 ± 0.03 [Bc]	10.58 ± 0.04 [Db]	6.30 ± 0.04 [Ab]	8.55 ± 0.04 [Cb]
Afternoon	7.63 ± 0.03 [Bb]	8.52 ± 0.12 [Da]	8.14 ± 0.04 [Cc]	7.03 ± 0.03 [Aa]
**Fuerte Quemado Site 1**				
Morning	15.92 ± 0.09 [Dc]	6.05 ± 0.03 [Ba]	5.08 ± 0.04 [Ab]	9.22 ± 0.09 [Cc]
Midday	14.11 ± 0.06 [Db]	6.43 ± 0.08 [Cb]	4.94 ± 0.05 [Ba]	2.89 ± 0.06 [Aa]
Afternoon	12.30 ± 0.06 [Da]	8.78 ± 0.02 [Cc]	7.22 ± 0.03 [Ac]	8.02 ± 0.06 [Bb]
**Fuerte quemado Site 2**				
Morning	12.27 ± 0.02 [Cb]	11.59 ± 0.07 [Bb]	4.41 ± 0.04 [Aa]	17.29 ± 0.08 [Dc]
Midday	14.67 ± 0.02 [Dc]	13.16 ± 0.14 [Cc]	11.75 ± 0.02 [Ab]	12.93 ± 0.04 [Ba]
Afternoon	9.42 ± 0.03 [Aa]	10.75 ± 0.09 [Ba]	12.52 ± 0.03 [Cc]	15.66 ± 0.07 [Db]
**Los Poleos Site 1**				
Morning	5.13 ± 0.02 [Ba]	13.44 ± 0.09 [Dc]	5.63 ± 0.02 [Cb]	4.97 ± 0.06 [Aa]
Midday	12.03 ± 0.04 [Dc]	8.56 ± 0.05 [Ba]	5.31 ± 0.05 [Aa]	8.78 ± 0.07 [Cb]
Afternoon	10.55 ± 0.06 [Db]	11.02 ± 0.20 [Cb]	6.77 ± 0.06 [Ac]	9.52 ± 0.05 [Bc]
**Los Poleos Site 2**				
Morning	10.41 ± 0.09 [Db]	6.61 ± 0.02 [Ca]	6.45 ± 0.03 [Bb]	5.41 ± 0.03 [Ab]
Midday	6.99 ± 0.03 [Ba]	10.84 ± 0.02 [Cb]	7.02 ± 0.06 [Bc]	6.06 ± 0.03 [Ac]
Afternoon	11.65 ± 0.11 [Cc]	13.81 ± 0.15 [Dc]	4.78 ± 0.03 [Ba]	4.13 ± 0.03 [Aa]

mg QE/g PM: milligrams of quercetin equivalents per gram of plant material. Data were analyzed by one-way ANOVA for each factor (season and time of day) independently, followed by Tukey's multiple comparison test ($p \leq 0.05$). Different capital letters in the same line indicate significant differences between seasons, and lowercase letters indicate significant differences between times of day.

Table S4. Content of reducing sugars in samples of *L. cuneifolia* collected in different seasons and at different times of the day.

Reducing sugars (mg GE/g PM)				
	Winter	Autumn	Spring	Summer
**Ampimpa Site 1**				
Morning	11.53 ± 0.08 ^{Cc}	12.48 ± 0.04 ^{Dc}	9.54 ± 0.04 ^{Bb}	7.76 ± 0.09 ^{Ab}
Midday	10.33 ± 0.03 ^{Cb}	10.44 ± 0.09 ^{Cb}	8.22 ± 0.02 ^{Ba}	7.73 ± 0.03 ^{Ab}
Afternoon	6.68 ± 0.03 ^{Aa}	8.86 ± 0.05 ^{Ba}	10.90 ± 0.04 ^{Cc}	6.68 ± 0.02 ^{Aa}
**Ampimpa Site 2**				
Morning	10.02 ± 0.03 ^{Cc}	15.17 ± 0.09 ^{Dc}	7.76 ± 0.03 ^{Ab}	8.69 ± 0.08 ^{Bb}
Midday	9.88 ± 0.01 ^{Bb}	11.01 ± 0.04 ^{Ca}	5.79 ± 0.07 ^{Aa}	9.95 ± 0.15 ^{Bc}
Afternoon	8.53 ± 0.02 ^{Ba}	13.53 ± 0.03 ^{Db}	9.19 ± 0.05 ^{Cc}	7.15 ± 0.11 ^{Aa}
**Tío Punco Site 1**				
Morning	8.63 ± 0.07 ^{Bb}	11.04 ± 0.04 ^{Db}	8.32 ± 0.04 ^{Ac}	9.68 ± 0.13 ^{Cb}
Midday	9.71 ± 0.02 ^{Cc}	10.13 ± 0.03 ^{Ca}	6.25 ± 0.04 ^{Aa}	8.08 ± 0.45 ^{Ba}
Afternoon	7.39 ± 0.05 ^{Aa}	13.50 ± 0.04 ^{Ba}	7.53 ± 0.07 ^{Ab}	7.36 ± 0.33 ^{Aa}
**Tío Punco Site 2**				
Morning	10.08 ± 0.01 ^{Ca}	12.23 ± 0.05 ^{Dc}	8.02 ± 0.05 ^{Ab}	9.77 ± 0.04 ^{Bc}
Midday	10.57 ± 0.09 ^{Dc}	10.22 ± 0.10 ^{Ca}	6.70 ± 0.08 ^{Aa}	9.43 ± 0.21 ^{Bb}
Afternoon	10.29 ± 0.02 ^{Bb}	11.04 ± 0.09 ^{Db}	10.69 ± 0.06 ^{Cc}	8.21 ± 0.03 ^{Aa}
**Fuerte Quemado Site 1**				
Morning	11.49 ± 0.04 ^{Dc}	9.22 ± 0.07 ^{Ab}	10.67 ± 0.03 ^{Cb}	7.76 ± 0.09 ^{Bc}
Midday	11.21 ± 0.08 ^{Db}	7.82 ± 0.10 ^{Ba}	9.28 ± 0.08 ^{Ca}	7.73 ± 0.03 ^{Aa}
Afternoon	10.32 ± 0.05 ^{Ba}	11.82 ± 0.09 ^{Cc}	12.20 ± 0.06 ^{Dc}	6.68 ± 0.02 ^{Ab}
**Fuerte quemado Site 2**				
Morning	10.65 ± 0.05 ^{Ba}	12.19 ± 0.08 ^{Db}	9.41 ± 0.05 ^{Aa}	11.17 ± 0.16 ^{Cc}
Midday	13.94 ± 0.06 ^{Dc}	11.71 ± 0.01 ^{Ca}	9.71 ± 0.06 ^{Ab}	9.89 ± 0.03 ^{Ba}
Afternoon	12.62 ± 0.09 ^{Db}	12.27 ± 0.06 ^{Cb}	11.62 ± 0.07 ^{Bc}	10.38 ± 0.05 ^{Ab}
**Los Poleos Site 1**				
Morning	7.82 ± 0.04 ^{Ba}	9.04 ± 0.06 ^{Db}	8.29 ± 0.06 ^{Ca}	5.68 ± 0.13 ^{Aa}
Midday	12.00 ± 0.02 ^{Dc}	8.13 ± 0.04 ^{Ba}	8.88 ± 0.05 ^{Cb}	7.77 ± 0.03 ^{Ab}
Afternoon	10.46 ± 0.02 ^{Cb}	12.25 ± 0.06 ^{Dc}	9.90 ± 0.07 ^{Bc}	7.79 ± 0.10 ^{Ab}
**Los Poleos Site 2**				
Morning	10.13 ± 0.02 ^{Dc}	7.14 ± 0.03 ^{Ba}	9.80 ± 0.03 ^{Cb}	5.83 ± 0.03 ^{Aa}
Midday	8.91 ± 0.07 ^{Ca}	10.87 ± 0.12 ^{Db}	7.66 ± 0.04 ^{Ba}	6.59 ± 0.07 ^{Ab}
Afternoon	9.86 ± 0.06 ^{Bb}	12.76 ± 0.08 ^{Cc}	10.04 ± 0.09 ^{Bc}	5.73 ± 0.07 ^{Aa}

mg GE/g PM: milligrams of glucose equivalent per gram of plant material. Data were analyzed by one-way ANOVA for each factor (season and time of day) independently, followed by Tukey's multiple comparison test ($p \leq 0.05$). Different capital letters in the same line indicate significant differences between seasons, and lowercase letters indicate significant differences between times of day.

Table S5. Protein content in *L. cuneifolia* samples collected in different seasons and at different times of the day.

Protein (mg BSA-E/g PM)				
	Winter	Autumn	Spring	Summer
**Ampimpa Site 1**				
Morning	65.45± 0.34 ^{Cc}	63.04 ± 0.56 ^{Bc}	75.87 ± 0.30 ^{Dc}	53.34 ± 0.51 ^{Ac}
Midday	55.59 ± 0.21 ^{Cb}	52.59 ± 0.18 ^{Bb}	55.95 ± 0.46 ^{Cb}	45.39 ± 0.10 ^{Ab}
Afternoon	36.75 ± 0.21 ^{Aa}	40.51 ± 0.48 ^{Ba}	42.51 ± 0.48 ^{Ca}	42.57 ± 0.10 ^{Ca}
**Ampimpa Site 2**				
Morning	84.04 ± 0.65 ^{Bc}	81.61 ± 0.45 ^{Ac}	98.73 ± 0.40 ^{Dc}	87.17 ± 0.54 ^{Cc}
Midday	62.40 ± 0.67 ^{Ab}	64.43 ± 0.12 ^{Bb}	74.00 ± 0.75 ^{Cb}	63.38 ± 0.32 ^{ABb}
Afternoon	47.91 ± 0.64 ^{Aa}	55.81 ± 0.39 ^{Ba}	66.97 ± 0.52 ^{Da}	57.70 ± 0.69 ^{Ca}
**Tío Punco Site 1**				
Morning	65.94 ± 0.22 ^{Bb}	62.84 ± 0.29 ^{Ab}	68.91 ± 0.40 ^{Cb}	66.84 ± 0.77 ^{Bb}
Midday	72.52 ± 0.27 ^{Bc}	66.40 ± 0.59 ^{Ac}	81.06 ± 0.89 ^{Cc}	73.46 ± 0.75 ^{Bc}
Afternoon	48.54 ± 0.59 ^{Ba}	46.70 ± 0.69 ^{Aa}	56.91 ± 0.28 ^{Da}	50.12 ± 0.46 ^{Ca}
**Tío Punco Site 2**				
Morning	67.24 ± 0.55 ^{Ab}	69.53 ± 0.41 ^{Bc}	73.68 ± 0.41 ^{Dc}	70.85 ± 0.31 ^{Cc}
Midday	56.13 ± 0.21 ^{Aa}	55.70 ± 0.49 ^{Ab}	58.53 ± 0.11 ^{Cb}	57.42 ± 0.38 ^{Bb}
Afternoon	68.43 ± 0.33 ^{Cc}	52.48 ± 0.38 ^{Aa}	51.96 ± 0.11 ^{Aa}	54.85 ± 0.29 ^{Ba}
**Fuerte Quemado Site 1**				
Morning	97.28 ± 0.39 ^{Dc}	78.10 ± 0.44 ^{Bb}	67.22 ± 0.53 ^{Ac}	79.98 ± 0.53 ^{Cc}
Midday	80.45 ± 0.97 ^{Db}	68.36 ± 0.30 ^{Ba}	59.44 ± 0.44 ^{Aa}	72.99 ± 0.90 ^{Cb}
Afternoon	78.63 ± 0.58 ^{Ca}	68.49 ± 0.85 ^{Ba}	62.54 ± 0.48 ^{Ab}	62.35 ± 0.70 ^{Aa}
**Fuerte quemado Site 2**				
Morning	70.69 ± 1.07 ^{Ab}	75.19 ± 0.61 ^{Bb}	91.33 ± 0.75 ^{Db}	81.86 ± 0.13 ^{Cb}
Midday	78.85 ± 0.96 ^{Ac}	84.47 ± 0.89 ^{Bc}	103.10 ± 1.04 ^{Cc}	83.27 ± 0.55 ^{Bb}
Afternoon	65.39 ± 1.04 ^{Ba}	70.98 ± 0.60 ^{Ca}	49.58 ± 0.71 ^{Aa}	67.22 ± 0.36 ^{Ba}
**Los Poleos Site 1**				
Morning	39.75 ± 0.42 ^{Aa}	63.42 ± 0.44 ^{Bc}	63.07 ± 0.32 ^{Bb}	66.26 ± 0.44 ^{Cb}
Midday	73.75 ± 0.79 ^{Cc}	59.55 ± 0.75 ^{Ab}	66.98 ± 0.14 ^{Bc}	73.99 ± 0.61 ^{Cc}
Afternoon	62.65 ± 0.35 ^{Db}	47.29 ± 0.26 ^{Aa}	57.48 ± 0.60 ^{Ca}	55.66 ± 0.68 ^{Ba}
**Los Poleos Site 2**				
Morning	85.86 ± 0.36 ^{Cc}	82.30 ± 0.27 ^{Bc}	71.93 ± 0.85 ^{Ac}	72.96 ± 0.14 ^{Ac}
Midday	72.32 ± 0.79 ^{Ca}	74.62 ± 0.69 ^{Db}	67.35 ± 0.13 ^{Bb}	55.94 ± 0.58 ^{Ab}
Afternoon	81.45 ± 0.49 ^{Db}	59.22 ± 0.36 ^{Ca}	54.51 ± 0.14 ^{Ba}	43.83 ± 0.47 ^{Aa}

mg BSA-E/g PM: milligrams of bovine serum albumin equivalent per gram of plant material. Data were analyzed by one-way ANOVA for each factor (season and time of day) independently, followed by Tukey's multiple comparison test ($p \leq 0.05$). Different capital letters in the same line

indicate significant differences between seasons, and lowercase letters indicate significant differences between times of day.

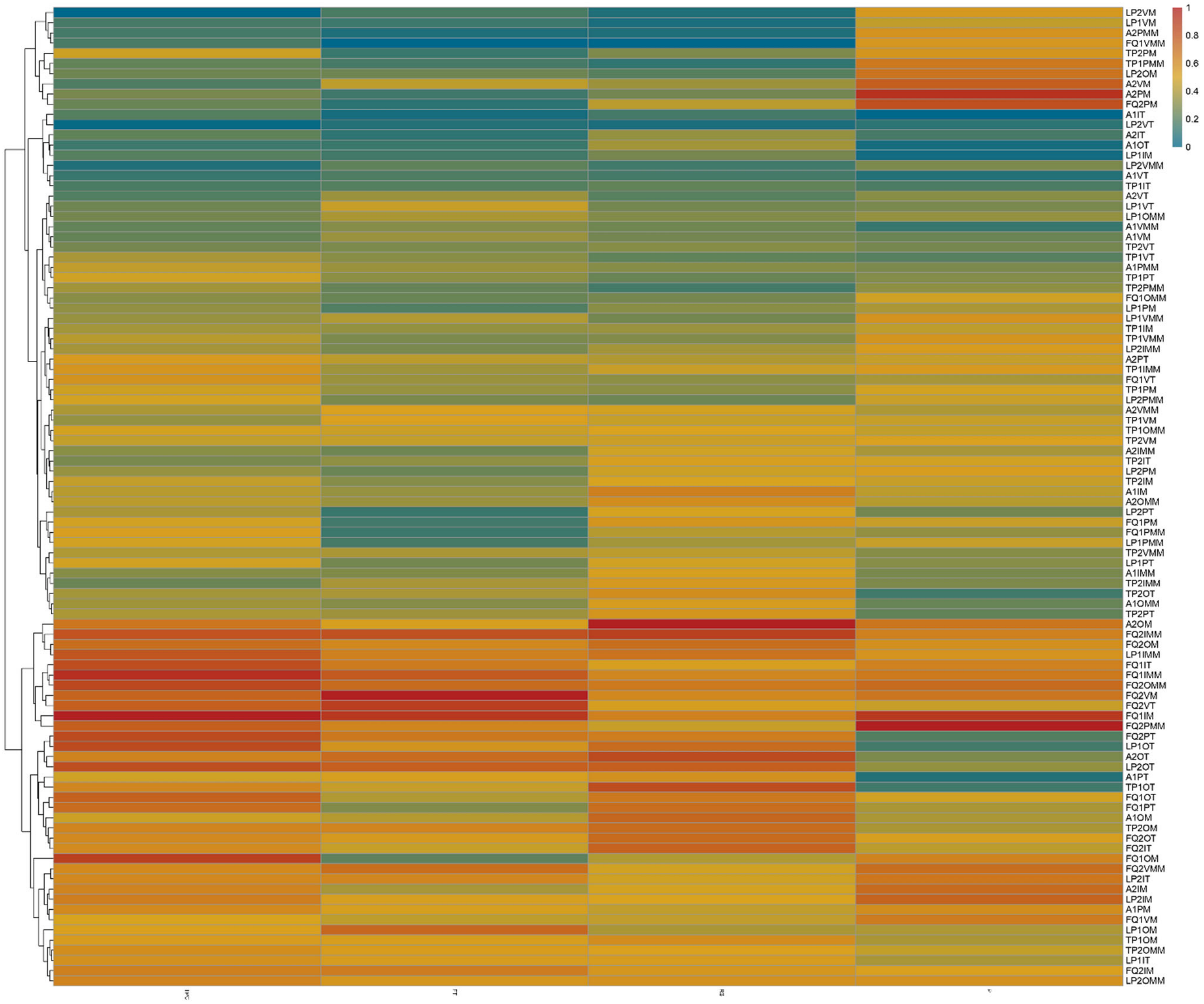

Figure S1. Heat map of the phytochemical composition of *L. cuneifolia* aerial parts collected at different sites. locations. seasons and times of day.
A1. Ampimpa site 1; A2. Ampimpa site 2; TP1. Tío Punco site 1; TP2. Tío Punco site 2; FQ1. Fuerte Quemado site 1; FQ2. Fuerte Quemado site 2; LP1. Los Poleos site 1; LPS2. Los Poleos site 2; I. winter; O. autumn; P. spring; V. summer; M. morning; MM. midday; T. afternoon; TPC. total phenolic compounds; TF. total flavonoids; RS. reducing sugars; SP. soluble proteins.
